# Partial hand and finger amputations in Sweden: an observational study of 6918 patients

**DOI:** 10.1186/s12891-024-07939-4

**Published:** 2024-10-19

**Authors:** Martin Magnéli, Michael Axenhus

**Affiliations:** 1 Orthopaedic Clinic, Danderyd University Hospital, Stockholm, Sweden; 2 Department of Clinical Sciences at Danderyd Hospital, Karolinska Institutet, Stockholm, Sweden; 3 Orthopaedic Clinic, Danderyd University Hospital, Entrévägen 2 182 68, Stockholm, Sweden

**Keywords:** Amputations, Incidence, Regional disparities, Upper Limb, Sweden

## Abstract

**Background:**

We aimed to use open source data to understand the incidence, trends, and regional differences of finger and partial hand amputations on a national level in individuals aged 15 or older in Sweden.

**Methods:**

We analyzed 6,918 patients aged 15 and older who had experienced finger and partial hand amputations. Incidence rates, trends, and regional disparities were assessed using negative binomial regression models and Student’s t-tests. Future trend prediction was performed using Poisson regression.

**Results:**

Finger amputations declined most, followed by partial hand and thumb amputations. Regional variations existed, with Stockholm having the lowest and Gotland highest incidence respectively. Overall, the incidence of finger, thumb and partial hand amputations in Sweden decreased slightly. Future trend analysis indicated decreasing incidence.

**Conclusion:**

Although, lacking in definition, publicly available data can be used for monitoring of finger, thumb, and partial hand amputation incidence on a national level. Sex, age, and regional differences were observed, suggesting the need for targeted interventions to address disparities and mitigate the burden of finger and partial hand amputations on affected individuals.

## Introduction

Finger and partial hand amputations are significant surgical procedures with profound implications for patients’ quality of life [1, 2]. The most common cause for finger and partial hand amputations are trauma, vascular disease and diabetes with age being a significant differentiator between causes [3, 4]. Traumatic injuries are common in the young population while disease related disability is more often the cause for amputation amongst the elderly [5]. Despite the profound impact of finger and partial hand amputation, fairly few studies have observed finger and partial hand amputation on a regional data level with existing studies mostly reporting national data level [6]. Recent studies have noted the need to examine geographical differences and sex disparities in limb amputations [7–9]. Understanding the incidence, trends, and regional disparities in limb amputations is crucial for informing healthcare policies and to guide future interventions [10, 11]. Using easily accessible data can allow researchers and stakeholders to observe trends and predict future outcomes. In this study, we conducted an observational population-based registry analysis utilizing open source data from the Swedish National Patient Register (NPR) spanning from 2008 to 2022. The NPR is a validated register for the administration of national-level healthcare. The NPR tracks all surgical procedures performed in Sweden and include all residents with a personal registration number [12]. Reporting to the NPR is mandatory for public healthcare making it suitable for analysis of surgical interventions. Furthermore, no specific upper extremity amputation register exists in Sweden.

The aim of this study is to investigate the incidence, trends, and regional disparities in partial hand, finger, and thumb amputations, among individuals aged 15 years and older in Sweden during a 15-year time period.

## Methods

### Aim

The aim of this study was to identify vulnerable patient population in regard to age and sex when it comes to partial hand and finger amputations in Sweden. Our secondary aim was to identify any potential regional differences in surgery incidence between regions in Sweden.

### Study design

This study is an observational population-based registry analysis, utilizing openly accessible data from the NPR spanning the period from 2008 to 2022. Incidence calculations relied on population data acquired from Statistics Sweden [13]. This study adheres to the STROBE guidelines for transparent reporting of observational studies [14].

### Setting

The Swedish National Health Service offers extensive healthcare coverage for all residents, encompassing emergency, inpatient, and outpatient services. The NPR monitors healthcare utilization among individuals with Swedish personal identification numbers, which serve as unique identifiers assigned to each citizen. These numbers facilitate healthcare monitoring and are fundamental for most citizen-government interactions. The NPR, alongside other Swedish registers, has undergone external validation, establishing it as a reliable data source for national-level healthcare expenditure analysis and population-based research [12].

### Data source

The NPR is a comprehensive database documenting patient treatment within the Swedish healthcare system. The register undergoes regular updates and is since June 2021 updated every month. Importantly, the NPR accommodates late-arriving data and post-publication corrections. All hospitals are mandated to contribute data, encompassing diagnosis and surgical procedure codes. For example, diagnoses have been coded according to the International Statistical Classification of Diseases version 10 (ICD-10) [15], since 1994, while surgical procedures adhere to NOMESCO’s classification system [16]. Integration into the NPR encompasses all orthopedic and hand surgery departments in Sweden performing amputation surgeries, ensuring comprehensive coverage.

### Partial hand and finger amputations

People aged 15 and above who underwent finger and partial hand amputation surgery from January 1, 2008, to December 31, 2022, were considered for inclusion. Only those with valid Swedish identification numbers were included to ensure precision. Each unique personal identification number was tallied once annually, per operation, and per geographical area. Amputation procedures were identified in the NPR utilizing NOMESCO codes. Various procedural codes were categorized based on the level of amputation (Table 1).

**Table 1:** Procedural codes and category of amputation levels. Each amputation level is associated with surgical procedure codes which indicate relevant NOMESCO codes and the defined amputation level per NOMECSO code

Amputation levels	Surgical procedure codes	Definition
Partial hand	NDQ11	Carpal
	NDQ12	Metacarpal
Thumb	NDQ13	Total thumb
	NDQ14	Partial Thumb
Finger	NDQ15	Total finger
	NDQ16	Partial finger

### Covariables

Incidence data were stratified by age, sex, 5-year age groups and region. Sweden is divided into 21 administrative regions, each responsible for healthcare and other services within its designated area.

### Statistics

Descriptive statistics were presented as total numbers and percentages. The ratio between the highest and lowest incidence rates was used for comparison. Students t-tests were utilized to compare difference in incidence between age groups, amputation level and sex. National and regional rates for all amputations and each level of amputation were calculated per 100,000 person-years, adjusting for age, sex, region, and year. Negative binomial regression models estimated annual changes in incidence rates over the 15-year period, adjusting for age and sex. We used Poisson regression to estimate incidence predictions up to 2030. Regional incidence was examined by comparing regional age-sex adjusted incidence of amputation rates. A p-value of less than 0.05 was considered significant. All calculations were performed using SPSS (Version 29.0).

### Human and animal rights

As this study utilized open source publicly available data, it was not subject to ethical review.

## Results

**Fig. 1:**
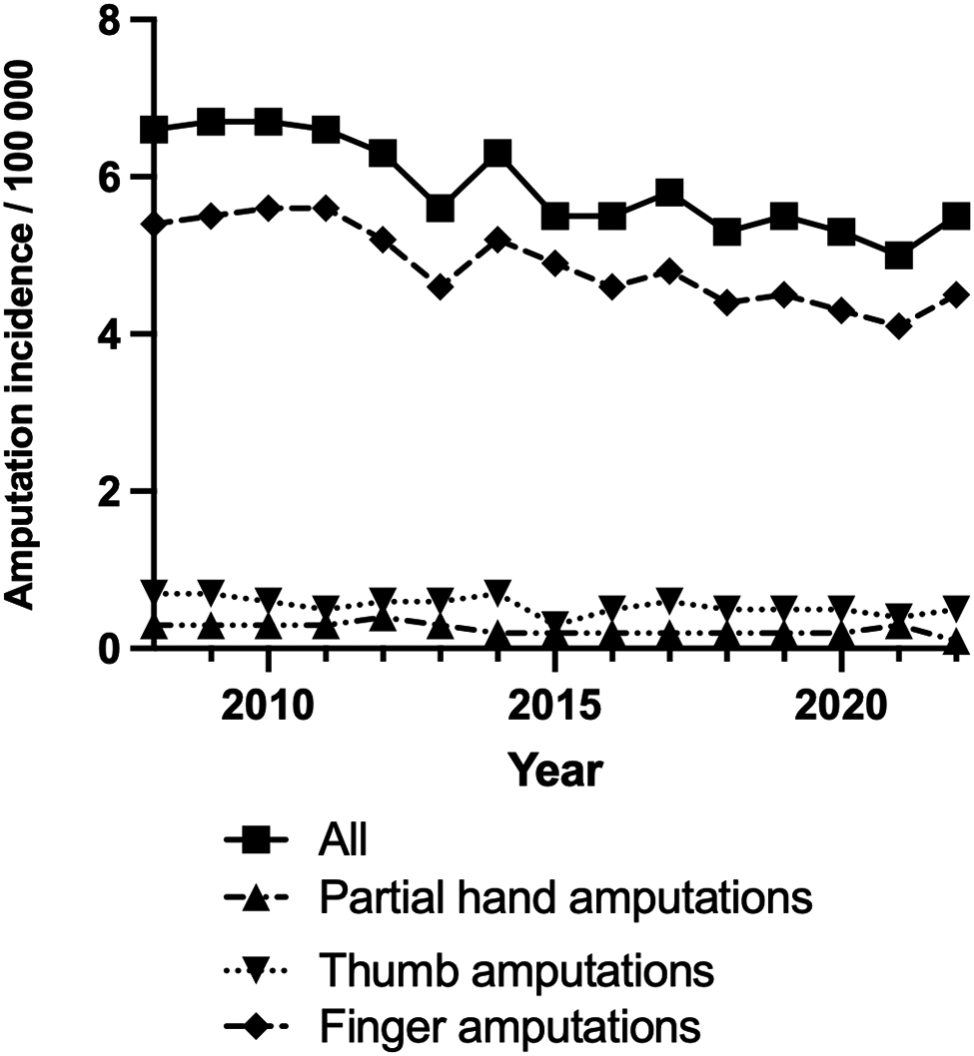
Trends in finger and partial hand amputations in Sweden during 2008–2022. Symbols indicate amputation levels

The trends in finger and partial hand amputations from 2008 to 2022 indicate a slight overall decrease in incidence rates. Partial hand amputations displayed minor fluctuations and maintained a consistent declining pattern with the change from 2008 to 2022 not reaching statistical significance (*p* = 0.53). Thumb amputations started at 0.7 per 100,000 inhabitants in 2008, decreasing slightly to 0.5 per 100,000 by 2011 and remaining around that level until 2022, although the change was not significant (*p* = 0.41). Finger amputations decreased significantly, (*p* = 0.021), from 5.4 per 100,000 inhabitants in 2008 to 4.1 per 100,000 inhabitants by 2022 (Fig. 1).

**Fig. 2:**
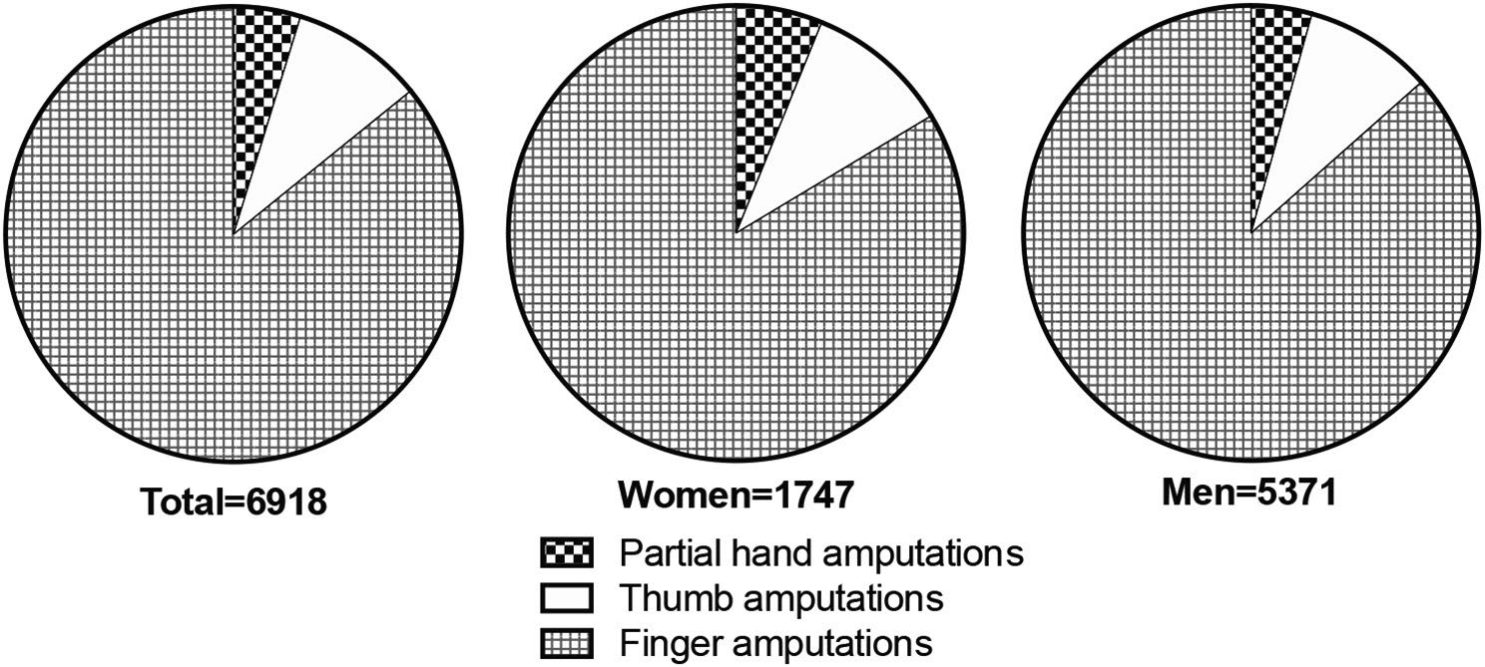
Distribution of partial hand, thumb and finger amputations between sex and anatomical site

Men underwent a higher number of all types of finger and partial hand amputations compared to women. Specifically, finger amputations were the most amputations common among both men (4509 cases) and women (1429 cases). The distribution between various finger and partial hand amputations were similar between men and women with no significant difference, (*p* = 0.12) (Fig. 2). The distribution of anatomical sites was similar for all years observed (data not shown).

**Fig. 3:**
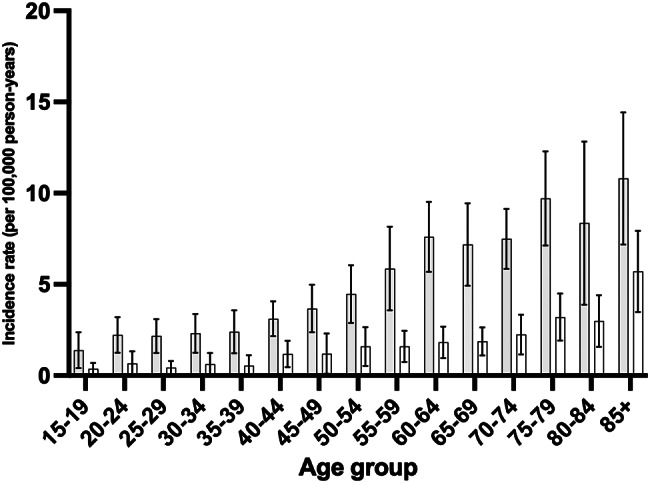
Age group distribution of partial hand, finger and thumb amputations. Grey indicates men and white indicate women. Error bars with 95% CI

The elderly population exhibited the highest incidence of amputations, with individuals under 60 years of age experiencing significantly fewer cases compared to older age groups, (*p* < 0.001). Additionally, men showed a significantly higher incidence of finger and partial hand amputations across all age categories when compared to women (*p* < 0.001) (Fig. 3). The age distribution was similar for all years observed (data not shown).

The overall incidence rate for all amputations was 14.3 per 100,000 person-years. Stratified by sex, women exhibited an incidence rate of 5.7 per 100,000 person-years, while men had a significantly higher rate of 8.7 per 100,000 person-years (*p* < 0,001). This difference was significant for partial hand (*p* = 0.007), thumb (*p* = 0.015), and finger (*p* < 0.001), amputations. The overall incidence rates for partial hand, thumb, and finger amputations were 0.5, 1.4, and 12.4 per 100,000 person-years, respectively. There was a slight decline in the incidence of all amputations over the study period, with an incidence change rate of 0.947 per year (95% CI: 0.789–1.105). Thumb amputations displayed the most pronounced decline, with an incidence change rate of 0.952 per year (95% CI: 0.938–0.967). Partial hand and finger amputations also exhibited decreasing trends, with incidence change rates of 0.978 (95% CI: 0.968–0.987) and 0.944 (95% CI: 0.875–1.014) per year, respectively (Table 2).

**Table 2:**
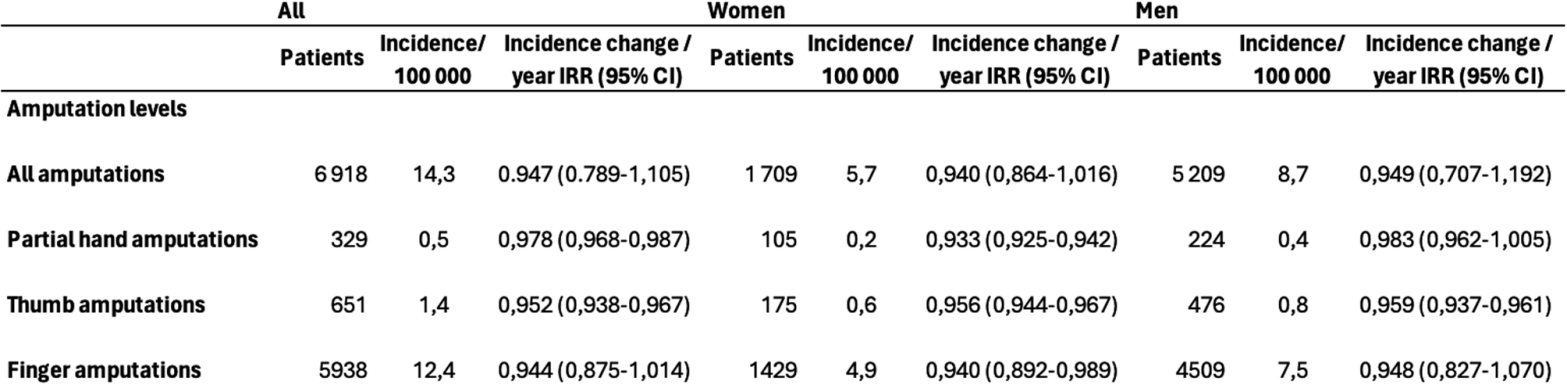
Incidence change rates (IRR), incidence rate and amputations per amputation level amongst men and women

**Fig. 4:**
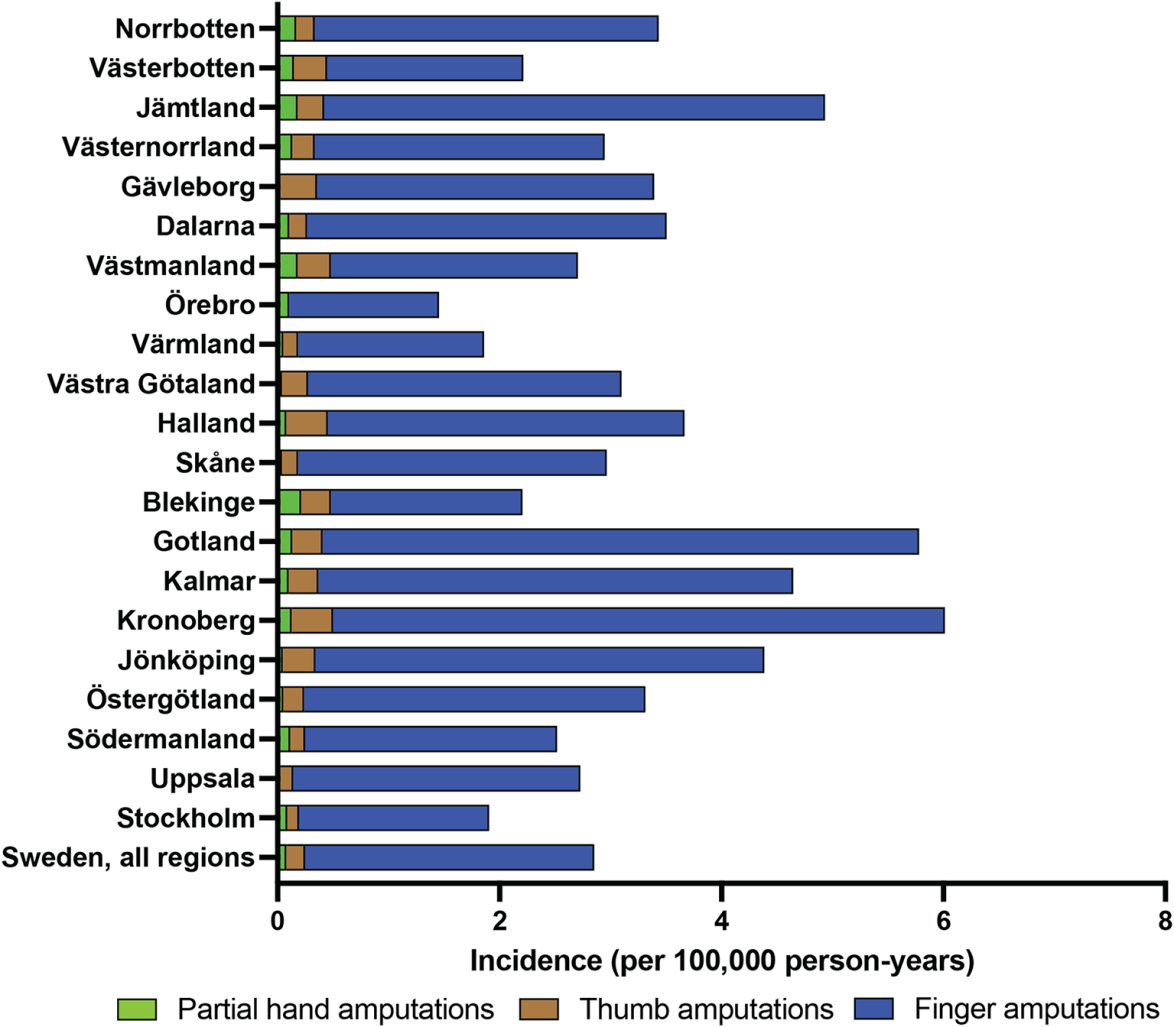
Regional distribution of finger and partial hand amputation per region and anatomical site during 2008–2022. Colors indicate amputation category

Regional variations were observed in the incidence of hand, finger, and thumb amputations. For instance, Stockholm reported the lowest overall incidence of 1.1 per 100,000 person-years, while Gotland had the highest at 9.7 per 100,000 person-years. Similarly, differences were noted in the incidence of partial hand, finger, and thumb amputations across various regions, with Kalmar exhibiting the highest proportion of finger amputations at 56% (Fig. 4).

**Fig. 5:**
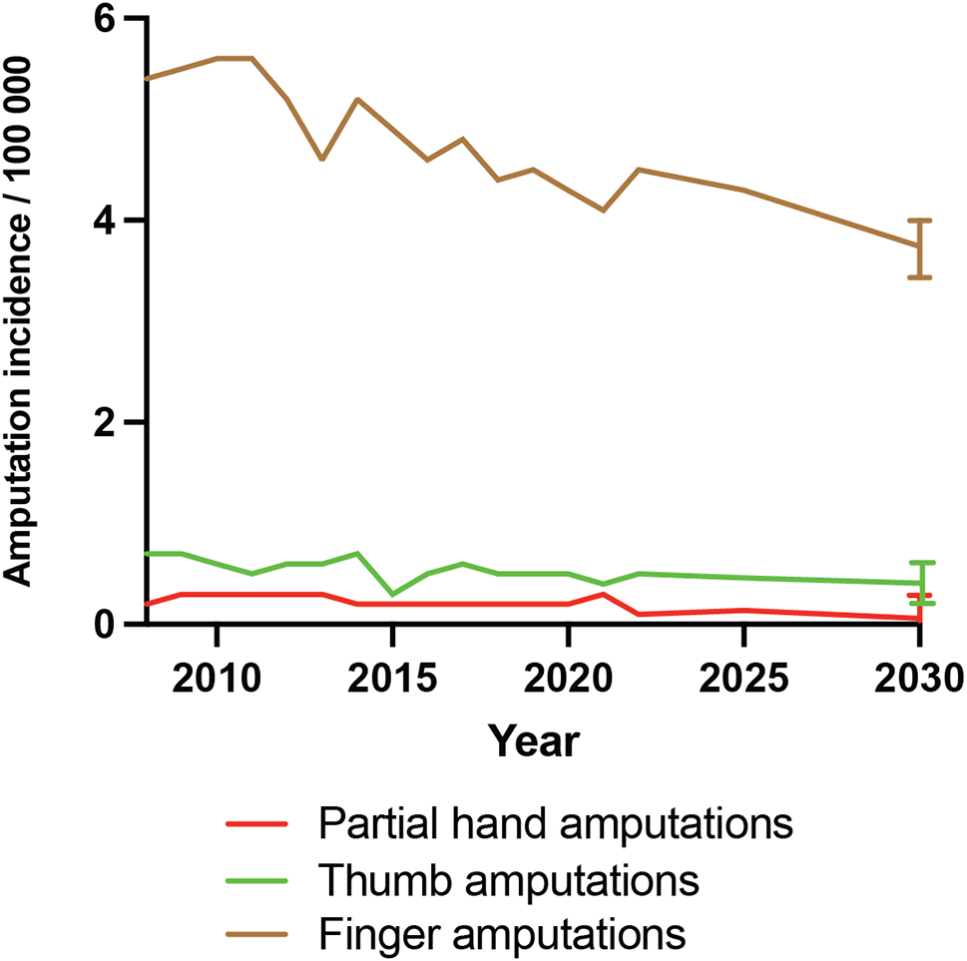
Future trend prediction of amputation incidence up until 2030. Error bars indicate 95% CI

Future trend prediction using Poisson regression indicate that by 2030, the overall amputation incidence will be lowered by 12% with finger and partial hand amputations decreasing more compared to thumb amputations (Fig. 5).

## Discussion

Finger, thumb and partial hand amputations can significantly impact individuals’ quality of life and pose challenges to healthcare systems globally. This study investigated the incidence, trends, and regional disparities in finger and partial hand amputations among individuals aged 15 years and older on a national population level basis in Sweden over a 15-year period.

We found a decrease in amputation rates which was driven by a decrease in finger amputation rates from 2008 to 2022. In total, finger amputation rates decreased by 24% during the study period. Sex disparities in finger and partial hand amputations were evident, with men undergoing more than three times as many amputations compared to women. Finger amputations were the most common among both men and women which is likely explained by a conservative approach to hand amputation [17, 18]. Although the distribution between various finger and partial hand amputations was similar between men and women, the overall incidence rate for all amputations was higher among men (8.7 per 100,000 person-years) compared to women (5.7 per 100,000 person-years). This finding aligns with previous research highlighting a higher prevalence of traumatic injuries, which often lead to upper limb amputations, among men [19, 20]. Men also generally experience higher incidence of secondary complications to digit injuries due to worse cardiovascular status subsequently necessitating amputation [21]. This was evident in our data with elderly men experiencing disproportionally more amputations than younger groups. For example, men aged 65 or older have approximate a three times higher incidence of amputation then men aged 25 years or younger. This likely indicate that hand surgeons will attempt flaps more often in younger populations, leading to lower amputation incidence in these groups.

Regional variations in the incidence of finger and partial hand amputations were notable, with disparities observed across different geographic areas of Sweden. Understanding the cause of these regional disparities could prove crucial for targeted interventions and resource allocation to address the underlying factors contributing to disparities in amputation rates. For example, Stockholm is a highly developed urban area, reported an incidence of 2 per 100,000 person-years while Gotland, a rural island region, reported 6 per 100,000 person-years. Gotland is a fairly isolated region consisting of two inhabited islands, there is one major hospital and no dedicated hand surgery clinic in the region. Patients in need of acute hand surgery are airlifted to neighboring regions for care. Similar challenges are present for other regions with high amputation rates such as the rural Jämtland region and sparsely populated Kronoberg region.

Regional variations in amputation rates may be linked to differences in treatment practices, and treatment preferences, as has been shown in other studies on limb amputations [22, 23]. Income disparity has been shown to correlate with disease comorbidity which predispose patient to amputations [24]. The three regions with the highest amputation rates—Gotland, Kronoberg, and Jämtland—all have some of the lowest average incomes compared to the Swedish national average. Furthermore, regional socioeconomic risk factors such as ethnicity, patients’ access to and utilization of preventive care, as well as their health education might influence amputation rates [25, 26] Future studies should focus on such risk factors as socioeconomic depravation, comorbidity, access to specialized hand surgery and local surgical trends to further elucidate the notable differences in amputations rates across Swedish regions.

Our findings suggest potential improvements in preventive measures, advancements in medical interventions, and enhanced rehabilitation strategies. However, the decrease in incidence clash with our findings of varying geographical incidence of finger, thumb and partial hand amputations. This might suggest that the decline in amputations might be driven by a few regions rather than an overarching national decline. Future trend prediction displayed a decreasing incidence in finger and partial hand amputations, a welcome finding.

Stakeholders might implement several strategies to further decrease the incidence of amputations. Targeted preventive care programs can be tailored to different age groups, with a focus on older adults at higher risk due to vascular diseases and diabetes. Considering the higher incidence of amputations among men, enhanced occupational safety training and cardiovascular health initiatives might prove useful to limit traumatic amputations and improve chances of reimplantation. Regions with higher amputation rates could also focus on improving access to specialized care through infrastructure enhancements, such as satellite clinics and telemedicine. Finally, the establishment of national guidelines for early identification and management of both acute and chronic conditions which leading to upper limb amputations might ensure standardized care across regions.

Several limitations should be considered when interpreting the findings of this study. The reliance on open-source registry data may introduce biases, including underreporting or misclassification, particularly due to regional coding variations. As a retrospective observational study, causality between observed trends and underlying factors, such as healthcare access, cannot be established. Poisson regression predictions also assume that current trends will persist, potentially overlooking changes in healthcare policies or population health. Additionally, the dataset did not allow for identifying specific causes of amputations. Nonetheless, this study demonstrates the value of open-source data in identifying national trends. To address the limitations of the retrospective design, future studies could use prospective cohort designs or case-control studies in order to identify key risk factors. Standardizing data collection across regions in longitudinal studies would also reduce inconsistencies and improve the reliability of the findings, the establishment of a quality upper extremity amputation register could be a useful tool in standardizing data collection.

In conclusion, this study provides a comprehensive analysis of the incidence, trends, and regional disparities in partial hand and finger amputations in Sweden over a 15-year period. Key findings include a significant decline in finger amputation rates, particularly among men and the elderly, and notable regional disparities. These findings highlight the need for targeted preventive measures, improved access to specialized care, and consideration of socioeconomic factors in healthcare planning. Future studies should aim to explore the underlying causes of these disparities and to develop interventions aimed at reducing the burden of amputations on affected populations.

## Data Availability

The data is available from the Swedish National Board of Health and Welfare [27]. Direct access link: https://www.socialstyrelsen.se/statistik-och-data/statistik/statistikdatabasen/. Clinical trial number: not applicable.

## Abbreviations

CI: Confidence Interval
ICD-10: International Statistical Classification of Diseases version 10
IRR: Incidence Change Rate
NPR: National Patient Register
NOMESCO: Nordic Medico-Statistical Committee
RECORD: REporting of studies Conducted using Observational Routinely-collected health Data
